# Study of Toxicity Assessment of Heavy Metals from Steel Slag and Its Asphalt Mixture

**DOI:** 10.3390/ma13122768

**Published:** 2020-06-18

**Authors:** Rui Hu, Jun Xie, Shaopeng Wu, Chao Yang, Dong Yang

**Affiliations:** State Key Laboratory of Silicate Materials for Architectures, Wuhan University of Technology, Wuhan 430070, China; hurui0907@whut.edu.cn (R.H.); wusp@whut.edu.cn (S.W.); hbyangc@whut.edu.cn (C.Y.); yangdhha@whut.edu.cn (D.Y.)

**Keywords:** steel slag, asphalt mixture, heavy metal contamination, the leaching behaviors

## Abstract

Steel slag has been used widely as an aggregate in road application, but it could pose a contamination risk for the environment due to considerable heavy metals (HMs). To explore the leaching behavior and contamination risk of HMs from steel slag and its asphalt mixture is of great significance. In this study, the physical-chemical features, batch leaching test and semi-dynamic test were conducted to determine the mobility capability and leaching characteristics of HMs. The results show that steel slag presents a low pollution risk in short-term leaching, whereas the cumulative release mass of Cd, Ni, As and Pb are more than or approach the limits, which indicates that steel slag exhibits environment impacts to a certain extent. Steel slag covered with asphalt binder results in As and Cu reduced by 3.64% and 4.83%. Diffusion is the main controlling mechanism of HMs in asphalt mixture and the mobility capability of most HMs were classed as “low mobility” (LI > 8). Asphalt stripping off can aggravate the release potential of HMs from asphalt mixture, but the pollution risk remains controllable.

## 1. Introduction

Waste solids are usually believed useless materials produced from all human industrial, commercial, building and demolition activities [1]. In the past, there were certain main ways to deal with waste solid including incineration and landfill, which can give rise to serious environment pollution. Hence, high-value reuse of waste solid materials plays a critical role in sustainable development. Additionally, large amounts of mineral materials have been consumed rapidly in road projects and construction. Attributed to the above, significant attempts have been made to study the engineering properties of various waste solids as raw materials in the construction industry, which is beneficial to relieve dependence on virgin mineral and divert waste materials from landfills. For instance, E. Tugrul et al. [2] studied waste marble as an ingredient in concrete, and indicated that the concrete which consists of waste marble instead of the coarse/fine aggregate, cement, and mixture material in certain rates exhibited higher strength. A Woszuk et al. [3] used zeolite materials as replacement of the filler in the warm-mix asphalt technology as an asphalt foaming additive, and found clinoptilolite could partially replace the traditional lime filler, without a negative impact on the asphalt mastic properties. Additionally, steel slag, characterized by high basicity, rich geometric features and excellent mechanical properties [4], has been justified its feasibility to replace aggregate in road engineering [5,6]. E Masad et al. [7] noted that the steel slag has the stronger bonding strength with asphalt binder in comparison to natural aggregates. Moreover, the water damage resistance, as well as the stiffness and fatigue life of steel slag based asphalt mixture also improved due to the vesicular and pitted structure of steel slag [8,9,10].

Different from the conventional hot-mixed asphalt (HMA), an asphalt mixture with steel slag does not need to consume substantial fossil fuels and mineral aggregates during raw material extraction. However, high-toxicity elements accumulate in the materials such as Pb, Cd and As [11,12,13], and could re-enter the surrounding environment though rainwater leaching, or dispersal by surface weathering under acidic conditions [14,15], which results in the deterioration of ecology, environment and biological diversity via the enrichment of heavy metals (HMs) in the food chain [16,17], and can lead to a variety of diseases, such as gastrointestinal diseases or neurological disorders [18,19]. In fact, roadway construction projects are beneficial to encapsulate the waste solids into an asphaltic matrix, which can prevent HMs’ release into the environment and reduce direct human exposure risk by minimizing the opportunity for contact [20,21,22]. However, owing to moisture damage, asphalt aging and vehicles load, the adhesion of asphalt mixture decreases, leading to asphalt stripping off from aggregate the [23,24]; meanwhile, those damages also aggravate the exposure risk and increase the pollution potential of steel slag.

Leaching is described as a complex process because many factors may influence the release of specific constituents from waste over a period of time. These factors include HMs concentration, pH, geochemical compositions, physical characteristics and contact time [25,26,27]. Studies have confirmed that the metals existing in exchangeable and acid-soluble form are regarded to be the easily available fractions, while the metals in reducible and oxidizable form are considered inert and can only leach out under extreme conditions [28,29]. Kierczak et al. [25] found that porous slag with increased reactive surface area releases more trace elements under surface weathering conditions. Ash et al. [26] stated that rainwater simulation solutions can leach more HMs from slag with time than deionized water, and slag heterogeneity and contact time are the prominent factors for the toxic metal release.

The contamination risk for water supplies from waste materials is typically assessed using leaching tests, which involve bringing tested materials into contact with an extractant under simulated acidic conditions in a laboratory. There are two main types of leaching test, namely the batch leaching test and the semidynamic leaching test. Due to the absence of information about the behavior of contaminants in the medium or long term, batch leaching tests used to assess the contamination risk and the short-term leachability of hazardous materials have been regarded as a conservative assessment of toxic elements’ release [30]. In semidynamic leaching tests (monolithic leaching test), on the contrary, waste materials encapsulated with asphalt binder or cement as an impermeable monolith are subjected to a leaching test in which the leachant is renewed with respect to time [31]. The methods can provide essential information about the leaching behaviors and the dominant leaching mechanism by allowing for the measurement of the time dependent release of trace elements and the calculation of observed elemental diffusivity [32].

In order to predict the leaching capability and contamination risk of HMs from steel slag and its asphalt mixture, an evaluation of the physical-chemical features of steel slag, a batch leaching test and a monolithic leaching test were performed to assess the release concentration of HMs. The objectives of this study are: (1) To determine the contamination potential of steel slag and its asphalt mixture of each HMs; (2) to assess the encapsulation ability of asphalt binder for HMs from steel slag; and (3) to determine the controlling leaching mechanism and observed diffusivity of HMs from the asphalt mixture.

## 2. Materials and Methods

### 2.1. Raw Materials

**Figure 1:**
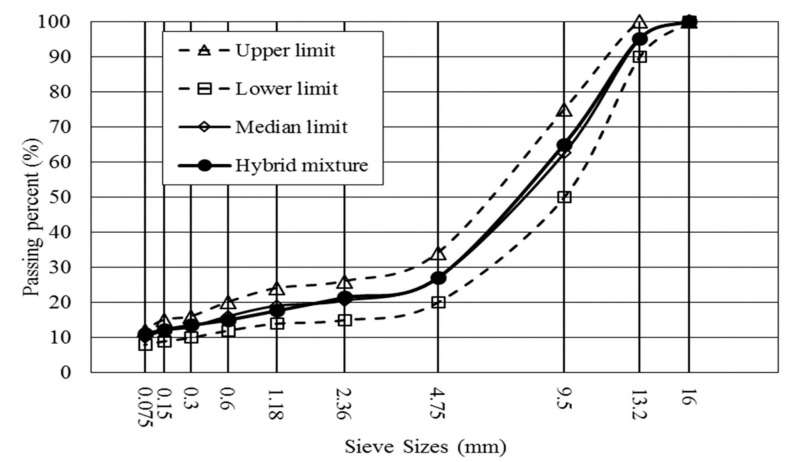
Hybrid gradation for asphalt mixture with basic oxygen furnace slag (BOFS) and basalt.

In this study, basic oxygen furnace slag (BOFS) was used, which was produced in Inner Mongolia, China. Basalt and commercial mineral filler were chosen from a local engineering project. The SMA-13 type of asphalt mixture with BOFS (coarse aggregate) and basalt (fine aggregate), defined as stone mastic asphalt with maximum dimension of aggregates of 13 mm, was designed by the Marshall method [33] and the optimum asphalt–aggregate ratio was determined to be 6.1%, where SMA-13 gradation was shown in Figure 1. BOFS, basalt and mineral filler in the mixture account for 79%, 11% and 10%, respectively, and basically properties of aggregate and asphalt binder are shown in Table 1 and Table 2.

### 2.2. The Physical-Chemical Features and Total Concentration of BOFS

The physical-chemical features of BOFS are characterized by microscopic morphology and mineral constituents. The microscopic morphology of the samples was characterized with a scanning electron microscope (SEM) (JSM-5610LV; JEOL Ltd., Tokyo, Japan). Mineral constituents were analyzed by X-ray diffraction (XRD) measurements produced from Bruker (Karlsruhe, Germany) with non-monochromated Cu Kα X-ray radiation.

Total concentration test: The total environmentally available concentration of elements in steel slag was assessed using the Environmental Protection Agency(EPA) method 3050b [34], partially modified. All samples of 0.2 g in powder form were placed in a bottle and mixed with 2 mL of HNO3 (65% purity), 6 mL of HCl (36% purity) and 1 mL of HF (38.2% purity). The bottles were put in the special digestion device, in which the temperature rose to 180 ± 10 °C. After 24 h, the bottles were taken out from the digestion device and heated on a hot plate. Two milliliters of HNO_3_ (65% purity) were added to the bottle until thickened to ensure hydrofluoric acid exhausted. Finally, deionized water was added to the extracted solution to make 10 mL of the analysis solutions after filtering insoluble residue of the samples. The result is shown in Table 3.

### 2.3. Leaching Tests

#### 2.3.1. Batch Leaching Test

EPA method 1311, the toxicity characteristic leaching procedure (TCLP), is a test that simulates contamination releases that are likely to arise when a material is stacked in the exposed condition. The procedure involves analyzing leachate samples to determine if the material is hazardous. The TCLP is a batch leaching test conducted at a “liquid to solid (L/S)” ratio of 20:1 (L/kg) with sample size reduced to pass a 9.5 mm sieve prior to test. Two different leachate extraction fluids were selected according to the samples’ alkalinity. Steel slag is a high-alkalinity waste solid, which is testified in the pre-experiment pH of > 5.0. In this study, about 50 g of the samples were weighed into polypropylene bottles, and 1 L of the TCLP reagent (0.1 M HAc, pH = 2.88 ± 0.05) was added. Next, the bottles were tumbled in a rotary extractor at 30 ± 2 rpm, and kept for 18 ± 2 h at room environment.

In order to overcome the conservative characters of TCLP in the assessment of the ecotoxicity, a progressive TCLP test [32] was conducted on the tested samples. This is attributed to the long-term leaching behavior and the objective contamination risk for HMs at acid or base environment. The test ran for a total of five progressive cycles, in which each cycle was the same as the TCLP test. After each extraction, the residues from the filtering process were returned to the extraction bottles to repeat the extraction using a fresh portion of the extractant.

#### 2.3.2. Monolithic Leaching Test

**Figure 2:**
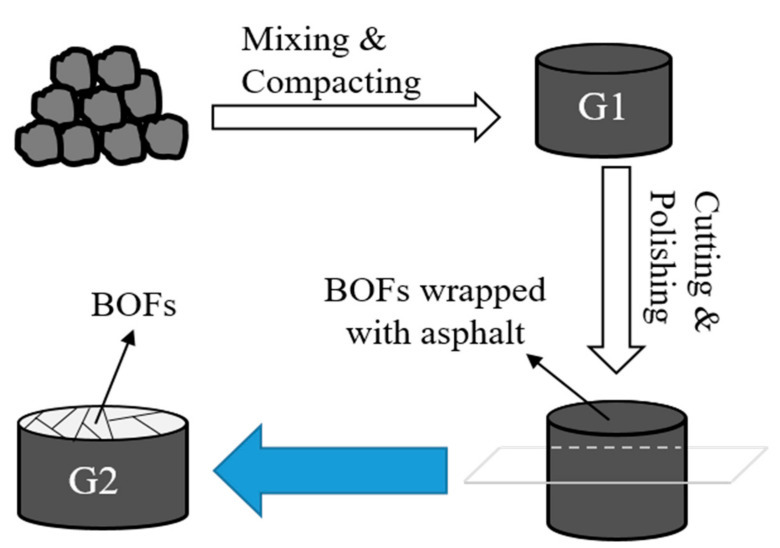
Preparation of the tested samples for the monolith test.

EPA method 1315 [34] was conducted on the asphalt mixture to study the long-term leachability and leaching mechanism of the samples in monolithic form. Method 1315 is a tank test where an intact sample is submerged in a vessel with the reagent, which consists of a blend of thick H_2_SO_4_ and HNO_3_ (weight ratio of 3:1; pH = 4.5), and the reagent is renewed at set time intervals (0.08, 1, 2, 7, 14 28, 42, 49 and 63 days), all method 1315 tests were conducted in triplicates. A liquid to exposed surface area ratio (L/SA) of 9 ± 1 mL reagent per cm^2^ of pavement area was used throughout the method 1315 test. Considering asphalt stripping off from BOFS, we cut off the surface of asphalt mixture and exposed the BOFS aggregate, for which the preparation process of tested samples is presented in Figure 2.

The equations for the cumulative mass release and observed diffusivity from the results of method 1315 can be calculated as follows in Equations (1) and (2). To calculate the observed elemental diffusivity for each interval ($D_{i}$) from the monolithic samples, an analytical solution derived from Crank [35] for diffusion from a cylinder into an infinite batch was used.

(1) $$D_{i}=\pi {\left[\frac{M_{ti}}{2\rho C_{0}\left(\sqrt{t_{i}}-\sqrt{t_{i-1}}\right)}\right]}^{2}$$

(2) $$D^{obs}=\frac{1}{n}\overset{n}{\underset{i=1}{\sum }}D_{i}$$

where: $D_{i}$ (m^2^/s) is mean observed diffusivity of a specific constituent for leaching interval; $M_{ti}$ (mg/m^2^) is mass released per unit area of the specimen during leaching interval; $t_{i}$ (s) is cumulative contact time after leaching interval; ρ (kg/m^3^) is the sample density;$\;C_{0}$ (mg/kg) is initial leachable content and $D^{obs}$ (m^2^/s) is mean observed diffusivity for the whole leaching test.

The leachability index (LI) is defined as the negative logarithm of the effective diffusion coefficient$\;D^{obs}$, which is calculated by Equation (3). The leachability index is used for comparing the relative mobility of the different contamination. According to the Canada environmental criterion for the use and disposal of stabilized/solidified wastes, wastes with a leachability index of <6.5 are considered to have high mobility, 6.5 < LI < 8.0 moderate mobility, and LI > 8.0 low mobility [20,36,37].

(3) $$\mathrm{LI}=-\log_{10}^{\left(D^{obs}\right)}$$

where: $D^{obs}$ is expressed in units of cm^2^/s.

### 2.4. Chemical Analysis

Chemical assessment of leachate is one test that is typically used to analyze the chemical toxicity of leachate samples. After the leachate samples are collected, they are typically filtered and the metals present in them are separated. The metals are typically quantified using an inductively coupled plasma atomic emission spectrometer (ICP-AES). The ecotoxicity and leaching risk of different heavy metals in BOFS are obtained in contrast to the environmental limits. In this chapter, the tested samples were made in triplicate to ensure the accuracy and that the content of heavy metals in the leachate was measured with average values.

## 3. Results and Discussions

### 3.1. Physical-Chemical Features and Total Concentration of Heavy Metals of BOFS

#### Physical-Chemical Features

**Figure 3:**
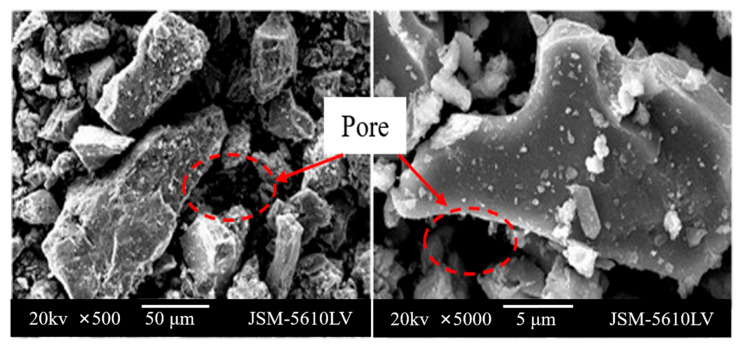
Scanning electron microscopy (SEM) images of BOFS.

The SEM observation of BOFS is presented in Figure 3. Some pores with different sizes are distributed on the surface of BOFS, which agrees with the fact that BOFS is a porous material. Additionally, it was obvious that the morphology feature of the surface of BOFS is very rich, which is one reason to form the abundant microspores. According to Z Jin et al. [38], the rich porosity of the surface of the mineral slag can absorb much water, causing long-lasting leaching for mineral slag, which aggravates the pollution risk of BOFS.

**Figure 4:**
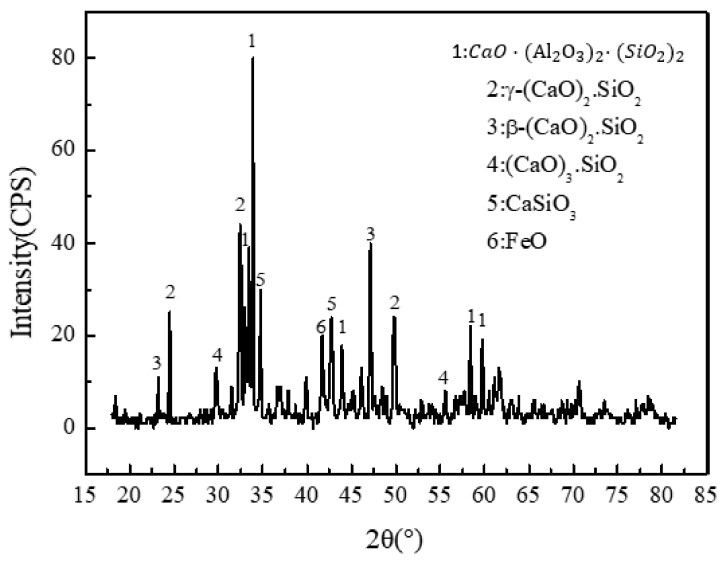
Mineral phases of BOFS.

X-ray diffraction provides clear clues for BOFS and the results showed that the dominant minerals were aluminosilicate $\left(\mathrm{CaO}\cdot {\left(Al_{2}O_{3}\right)}_{2}\cdot \left(SiO_{2}\right)\right)$, silicate minerals ${\left((\mathrm{CaO}\right)}_{x}\cdot {\left(SiO_{2}\right)}_{y})$, and FeO, as seen in Figure 4. Additionally, other complex compounds, including toxic metals such as hashemite ($BaCrO_{4}$) and crocoite ($PbCrO_{4}$) [39], in steel slag were not exhibited because they are beyond the limits of detection.

Table 3 shows the total concentrations of Cd, As, Cu, Pb, Cr^6+^, Zn and Ni in BOFS, basalt and mineral filler and the result presents that the content of heavy metals in basalt and mineral filler is far lower than in BOFS. According to the weight percentage of these materials, BOFS dominates. Additionally, due to the fine particle size of basalt and the mineral filler, the thicker asphalt membrane on the surface of the both materials could result in the lower heavy metal release. So, we regarded BOFS as the main source of heavy metal release from the mixture.

BOFS contains abundant toxic elements, defined as potential contamination of waste material. For the content of heavy metals, Pb and Cr^6+^ are considered high, followed by Zn, Cd and Cu, and As an Ni are deemed as low.

### 3.2. Evaluation of the Leaching Toxicity of BOFS and its Asphalt Mixture

#### 3.2.1. TCLP Test

**Figure 5:**
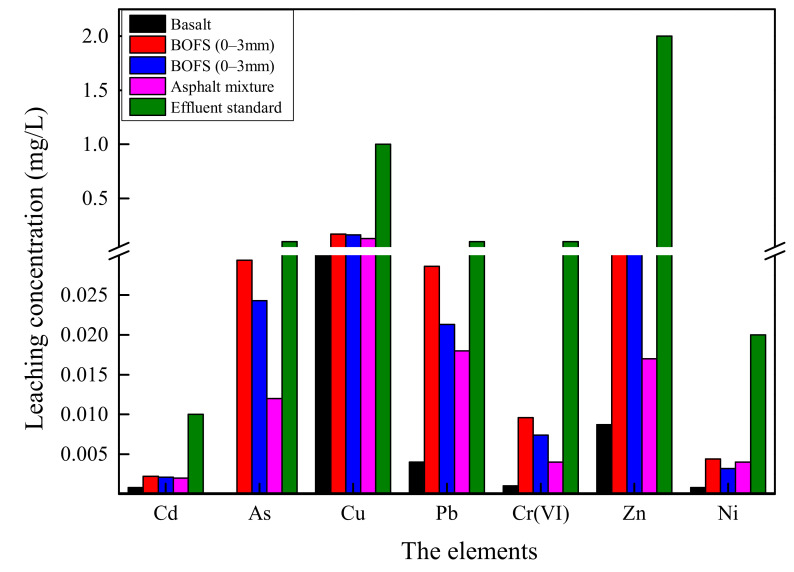
The leaching results of toxicity characteristic leaching procedure (TCLP) test from BOFS, basalt and asphalt mixture.

The batch leaching test allows the acidic extractant to erode and destroy the internal structure of BOFS, which causes HM release. In this experiment, the TCLP test was used to assess the contamination risk of HMs to aggregate and its asphalt mixture, and the results are summarized in Figure 5. The leaching concentration of HMs of BOFS with 0–3 mm range are higher than that with 3–5 mm range, but the values are in the same magnitude without large difference. This indicates that BOFS with small size, which is characterized by abundant pores and larger contact area, has greater capability for HM release. The leaching content of heavy metals in basalt is far lower to BOFS. Asphalt mixture presents a lower leaching content of HMs than BOFS, which shows that asphalt binder can mitigate HM release and that the asphalt mixture presents lower pollution risk. Additionally, measurements from the TCLP test are no more than the environmental limits. On the basis of the results, it is concluded that BOFS has no heavy metal contamination in short-term leaching and asphalt mixtures can be used for safe disposal for BOFS.

#### 3.2.2. Progressive TCLP Test

**Figure 6:**
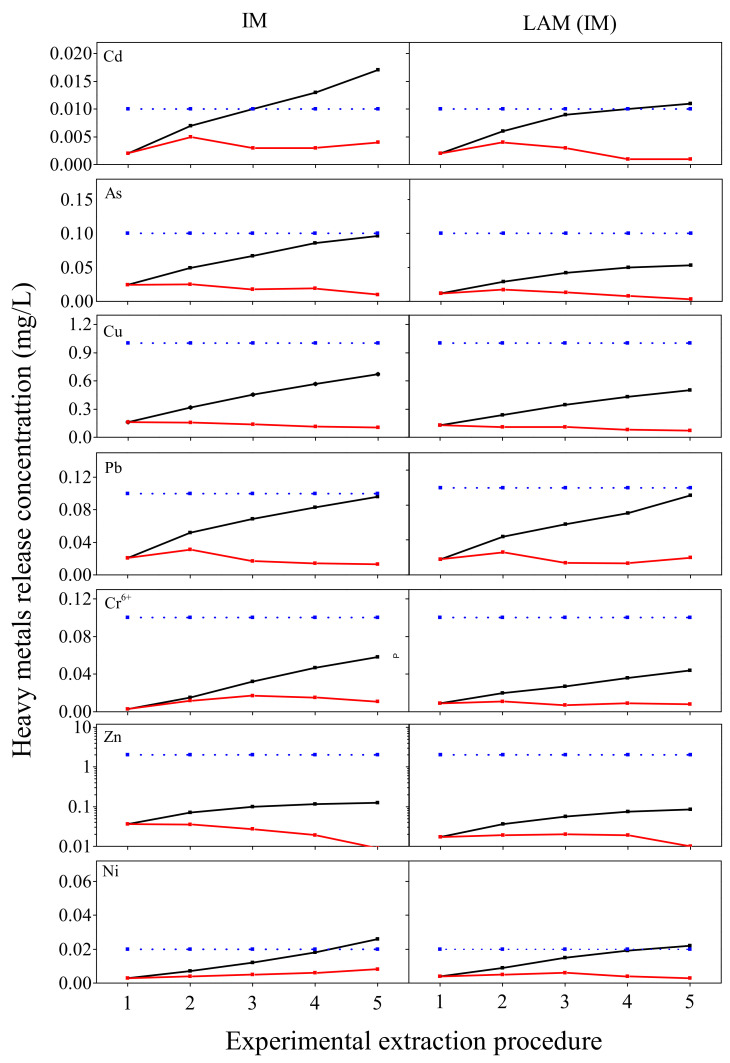
Release concentrations of the leaching events in progressive TCLP test; the red line represents concentration of different leaching cycles; the black line is cumulative leaching concentration and the blue line is Class IV limit of China Nations Environmental Quality Standards for surface water.

The results from progressive TCLP test may give a first view of the leaching behavior of HMs from BOFS and its loose asphalt mixture (LAM). As shown in Figure 6, the concentrations of As, Pb, Cr^6+^ and Zn of BOFS reached the maximum at the second cycle and Cu was in peak at the first cycle, then decreased along with proceeding of test in leachates. The contents of Cd had no remarkable difference with leaching cycles. Combined the cumulative release mass of HMs and the environmental limits, it can be found that the cumulative contents of some HMs has had an impact on environment despite the safe concentration at each extractant. The cumulative concentrations of Cd and Ni from BOFS were higher than the limits. On the basis of the results, a conclusion can be formed that BOFS can cause heavy metal pollution in long-term leaching.

In comparison with BOFS, the heavy metal contamination of its loose asphalt mixture is reduced remarkably. For LAM, the cumulative concentrations of Cd and Ni reached the limits, which are lower than the contamination level of BOFS. The cumulative concentration of Pb was satisfied with the limits, where it exceeds the limits in BOFS. It manifests that asphalt binder wrapping in the surface of BOFS can reduce effectively the HMs release and decrease the contamination level. The results correspond with J. Xue et al.’s study [40], where the leaching concentration is distinct decrease after being capsulated by asphalt in SMA mixture for BOF aggregate. But the leaching value of asphalt mixture in his research is higher, which is due to the decrease of adhesion of BOFS with asphalt in service life, resulting in the greater heavy metals release from asphalt mixture.

**Figure 7:**
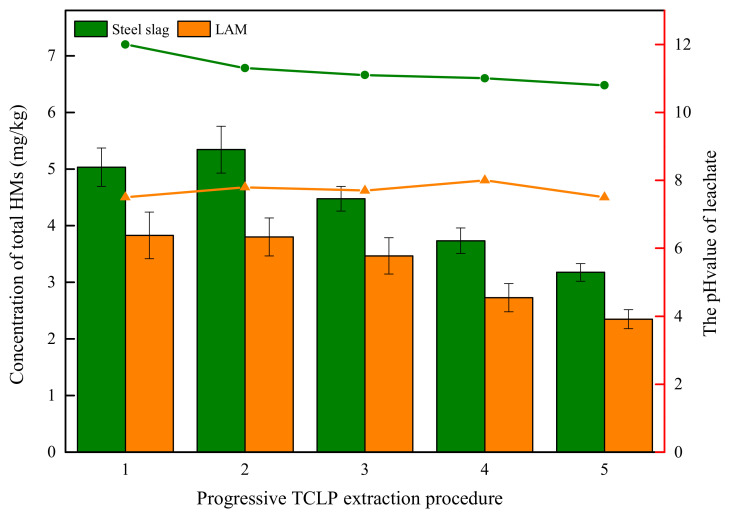
Concentration of all HMs and the pH value in progressive TCLP test.

The leaching features of all HMs in BOFS and its asphalt mixture are presented in Figure 7. The maximum leaching content of all HMs in leachates occurred the first two times, then decreased. The essence of acid leaching is the reaction of H^+^ ions with acid-buffer components (including mineral constituents of heavy metals) in slags [41]. At the beginning of leaching, H^+^ ions of extractant exchanged with the toxic metals of BOFS, resulting in the release of large amounts of HMs and then the reduction of HM release with the content of HMs in BOFS decreasing. Additionally, the release concentration of all HMs in BOFS was more than that of its asphalt mixture at each cycle extraction.

The pH value of leachate has a significant positive correlation with the content of elements after the leaching events, in which higher pH value implies the higher content of elements in leachate. The pH value of BOFS leachate ranged from 10 to 12, whereas the pH value of LAM leachate varied from 6 to 8. This could be due to the fact that asphalt binder has the encapsulation effect on BOFS to hinder acid-buffer components release into extractant, which contributes to the lower pH value after leaching events. We conclude that engineering project can decrease the contamination risk through BOFS encapsulated by asphalt binder.

#### 3.2.3. Cumulative Leaching Rate

Cumulative leaching rate (r) is often used to evaluate the leaching capacity of heavy metals in solid waste. It is defined as the ratio of the cumulative release amount of metal concentrations leached out ($C_{i}$) to the total metal concentrations ($C_{t}$) in solid waste materials and can be calculated according to the following Equation (4).

(4) $$r=\sum \frac{C_{i}}{C_{t}}\times 100\%$$

**Figure 8:**
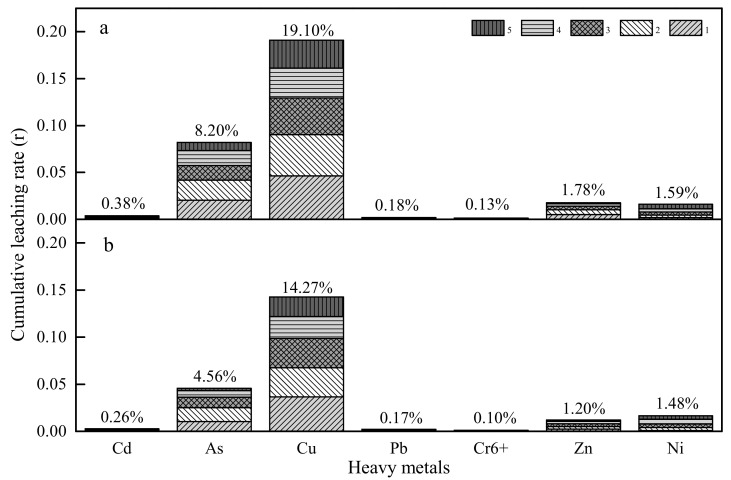
The cumulative leaching rate (r) (**a**) BOFS; (**b**) loose asphalt mixture (LAM).

Cumulative leaching rate (r) is considered as an important index to assess the leaching capability of HMs from BOFS and loose asphalt mixture, and quantifies the encapsulation effect of asphalt binder for HMs release, which is dependent on total concentration of HMs and leaching content from BOFS. As seen from Figure 8, the cumulative leaching rates of As, Cu, Zn and Ni were 8.20%, 19.10%, 1.78% and 1.59%, respectively, while the r of Cd, Pb and Cr^6+^ was less than 1% that from BOFS.

The results from the evaluation of leaching behavior of HMs from loose asphalt mixture based on BOFS are shown in Figure 8. It is very important to mention that the amounts extracted are far less than those extracted from BOFS. Especially, the cumulative release mass of As and Cu from LAM reduced by 3.64% and 4.83% respectively. The leaching content of other metals did not decrease markedly due to the feebleness of the leaching capability of BOFS.

### 3.3. The Release Characteristics of HMs from Asphalt Mixture

Steel-slag-based asphalt mixture is utilized in road engineering and exhibits excellent pavement performances. R Hu et al. [42] found asphalt mixtures with BOF slag present superior moisture resistance to that with limestone and andesite. J. Xie et al. [8] found BOF slag showed superior rutting resistance with asphalt to basalt. In this chapter, we assessed asphalt mixture with BOF slag from environmental implication, where EPA method 1315 was conducted to evaluate the contamination risk of BOF-slag-based asphalt mixture as well as the mixture with asphalt stripped off (G1 and G2).

#### 3.3.1. The Characteristics of HMs Release from the Monolithic Sample

**Figure 9:**
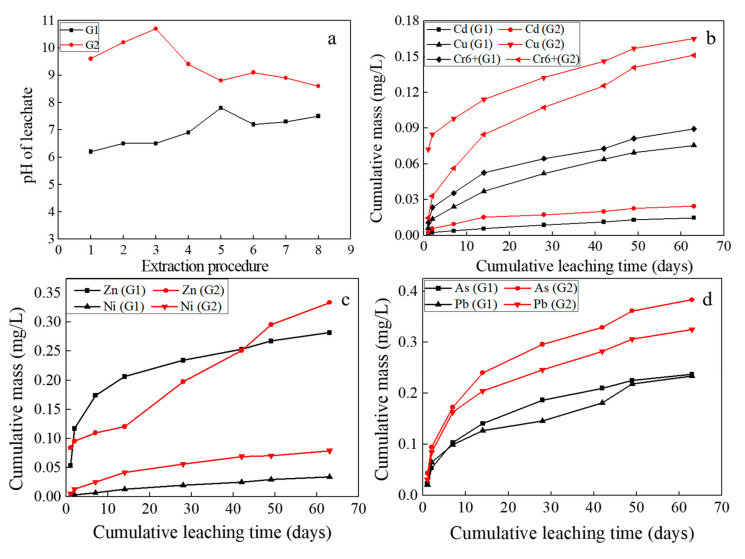
Evolution of pH and HMs from the monolithic asphalt mixture and that with asphalt stripped off (G1 and G2). (**a**) The pH value of the leachate, (**b**) The cumulative mass of Cd, Cu and Cr^6+^ in the both samples, (**c**) The cumulative mass of Zn and Ni in the both samples, (**d**) The cumulative mass of As and Pb in the both samples.

Figure 9 displays the pH values and the elements’ release for the asphalt mixture samples in the 1315 method test. As seen from Figure 9a, the pH values of G1 began at a pH of 6.2 (day 1.04) and ended at a pH of 7.5 (day 63), while the pH values of G2 began at a pH of 9.7 and ended at a pH of 8.5. The pH values of the leachate reached the maximum at day 28 and day 7, respectively 7.2 (G1) and 10.5 (G2). The encapsulation effect of asphalt on BOFS hampers the acid-buffer components and HMs release, which results in low pH of leachate in G1 and prolongs the release time. Additionally, the leaching potential of HMs release from asphalt mixture increase after asphalt stripping off. As shown in Figure 9b–d, the cumulative release mass of HMs in G2 exceeded that in G1, among which Cu and Ni were more than 100%, followed by Cd, As and Cr^6+^ over 50%, and Pb was lowest, around 30%.

#### 3.3.2. The Mechanisms of HMs Released from the Monolithic Sample

To determine the release mechanisms of different elements, an approach that a graph of the cumulative mass release ($M_{i}$) and the cumulative leaching time on a log-log scale was employed. The slope of the line was used as an indicator to assess the leaching mechanism of different metals in asphalt mixture. For diffusion to be indicated as the governing release mechanism, the slope of the line needs to fall between 0.35 and 0.65. A slope of greater than 0.65 indicates dissolution, while depletion is defined as the predominant form of release for a slope less than 0.35 [20,37].

Table 4 presents the slope and relevancy (R^2^) of lines of the log-log comparison of the cumulative release mass ($M_{i}$) and the cumulative leaching time (t). The R^2^ values of all fitting lines were greater than 0.94, which shows a good fit of the regression to the measured data points. For G1, the slopes of Cd, As, Cu, Pb and Cr^6+^ distribute between 0.35 and 0.65, which are indicated as diffusion-controlled mechanisms, while the slope of Zn is less than 0.35, deemed as depletion, and the slope of Ni is more than 0.65, considered as dissolution. For G2, the release mechanism of Cd, As, Pb, Cr^6+^ and Ni are indicated as diffusion-controlled, and Cu and Zn are found to be depletion-controlled. The above results indicate that diffusion-controlled is the main release mechanism for HMs from asphalt mixtures, which also can be confirmed in other studies [43,44], and the release mechanism of heavy metal could change after stripping off asphalt from BOFS.

#### 3.3.3. Observed Diffusivity (D^obs^) and Leachability Index (LI)

Mean diffusivity and leaching index are used to assess the mobility of heavy metal from asphalt mixture, where the results are summarized in Table 5. The D^obs^ values of HMs from G1 varied from 2.28 × 10^−7^ to 2.09 × 10^−11^ (cm^2^/s). When classified using the LI, these values fell between 6.6 and 10.7, while the D^obs^ values of HMs from G2 ranged from 3.78 × 10^−6^ to 3.27 × 10^−12^ (cm^2^/s). When classified using the LI, these values fell between 5.4 and 11.5. The LI of HMs from G1 are more than 8, described as low mobility, except As (6.5 < LI < 8) known as moderate mobility. For G2, the LI of Cu, Pb, Cr^6+^, Zn and Ni are higher than 8, defined as low mobility, while Cd (LI = 7.1) is considered as moderate mobility and As (LI = 5.4) is regarded as high mobility. Based on the results, we conclude that most elements of BOFS were immobilized in the monolith, with low mobility and controlled utilization, but the mobility and pollution potential could augment after stripping off asphalt.

## 4. Conclusions

The leaching behavior and contamination potential of BOFS and its asphalt mixture were determined through the evaluation of the physical-chemical features of BOFS, the TCLP test, progressive TCLP test and the monolith test. BOFS has substantial amounts of HMs, while it is a high-porosity and complicated phase constituent material. The TCLP test result showed that it has no environmental issues in short-term leaching, but it can cause heavy metal pollution in long-term leaching, which could reflect the fact that the cumulative release mass of Cd and Ni in the progressive TCLP test is more than the environmental limit. Asphalt binder can mitigate heavy metal release from BOFS by forming a matrix monolith. From the leaching rate, asphalt binder lessened the leaching amount of As and Cu in BOFS by 4%–5%, while other elements did not remarkably reduce due to the low leaching rate in BOFS.

From the results of method 1315, HMs in asphalt mixture with BOFS (G1) exhibit low mobility (LI > 8), considered as safe disposal, expect As (6.5 < LI < 8), defined as moderate mobility, but asphalt binder stripping off can increase the mobility of heavy metal. As seen from G2, the majority of elements remain to be low mobility, but As (LI = 5.4) is regarded as high mobility and Cd (LI = 7.1) is deemed as moderate mobility. Additionally, the “diffusion controlled” is the main release mechanism for heavy metal in G1 and G2, except Zn from G1 and Cu and Zn from G2 which are depletion controlled. Therefore, BOFS as an aggregate in asphalt mixtures can reduce heavy metal contamination caused by direct exposure to the environment, but asphalt binder stripping off can increase the pollution potential and mobility.

## Figures and Tables

**Table 1:** Basic properties of BOFS and basalt.

Indexes	Standard	Hybrid Mixture		Requirements in China
		BOFS (Coarse Aggregate)	Basalt (Fine Aggregate)	
Apparent density (g/cm^3^)	ASTM C127	3.66	2.75	Min 2.6
Water absorption (%)	ASTM C127	1.18	0.3	Max 3
Crush value (%)	ASTM C127	12.9	17.1	Max 22
Los Angeles abrasion (%)	ASTM C131	8.3	16.8	Max 22
Free lime content (%)	BS EN1744-1	2.3	N/A	Max 3

**Table 2:** Basic properties of asphalt binder (AH-70).

Indexes	Standard	Measured Values	Requirements in China
Penetration at 25 °C (0.1 mm)	AASHTO T228	63	60–80
Softening point (°C)	AASHTO T53	48	Min 46
Ductility at 15 °C (cm)	AASHTO T51	>160	Min 100
rotational viscosity at 135 °C (Pa.s)	AASHTO T201	0.596	Max 3
Flash point (°C)	AASHTO T48	413	Min 260

**Table 3:** Initial Concentration of heavy metals (HMs) of BOFS, basalt and mineral filler.

Elements	Initial Concentration (mg/kg)					
	BOFS		Basalt		Mineral Filler	
	Avg ± Stdev	CV (%)	Avg ± Stdev	CV (%)	Avg ± Stdev	CV (%)
Cd	89.65 ± 4.12	4.6	15.14 ± 0.85	5.6	11.34 ± 0.15	1.3
As	23.13 ± 2.37	10.3	ND	-	ND	-
Cu	70.38 ± 2.84	4.0	12.07 ± 0.46	3.8	12.65 ± 0.67	5.3
Pb	978.41 ± 13.47	1.4	28.34 ± 0.37	1.3	22.56 ± 1.34	5.9
Cr^6+^	851.38 ± 27.18	3.2	16.85 ± 1.37	8.1	24.73 ± 2.45	9.9
Zn	141.05 ± 14.33	10.2	ND	-	8.38 ± 0.67	8.0
Ni	32.25 ± 1.60	5.0	2.5 ± 0.12	4.8	6.53 ± 0.44	6.7

CV: The coefficient of variation (Stdev/Mean), %; ND: Below detective content.

**Table 4:** Release mechanism of HMs from G1 and G2.

Elements	G1		G2	
	Slope	R^2^	Slope	R^2^
Cd	0.527	0.979	0.51	0.952
As	0.528	0.967	0.489	0.947
Cu	0.583	0.988	0.195	0.982
Pb	0.512	0.972	0.503	0.948
Cr6+	0.472	0.97	0.524	0.964
Zn	0.346	0.987	0.331	0.952
Ni	0.744	0.996	0.611	0.967

**Table 5:** Observed diffusivity and leachability index of HMs.

Elements	G1		G2	
	D^obs^ (cm^2^/s)	LI	D^obs^ (cm^2^/s)	LI
Cd	5.01 × 10^−11^	10.3	8.64 × 10^−8^	7.1
As	2.28 × 10^−7^	6.6	3.78 × 10^−6^	5.4
Cu	2.23 × 10^−9^	8.7	3.27 × 10^−12^	11.5
Pb	8.14 × 10^−11^	10.1	5.11 × 10^−11^	10.3
Cr^6+^	2.09 × 10^−11^	10.7	1.07 × 10^−10^	10
Zn	9.57 × 10^−9^	8.1	4.77 × 10^−10^	9.3
Ni	1.69 × 10^−9^	8.8	5.71 × 10^−10^	9.2
